# Active clinically isolated cardiac sarcoidosis with inactive pulmonary sequelae: incremental diagnostic value of multimodality imaging: a case report

**DOI:** 10.1093/ehjcr/ytag545

**Published:** 2026-08-17

**Authors:** Leonardo Briceño Loaiza, Ximena Celi Loaiza

**Affiliations:** Department of Cardiac Imaging, Solca Hospital, Av. Salvador Bustamante Celi y Agustín Carrión, Loja 110104, Ecuador; Department of Cardiac Imaging, Hospital Moinhos de Vento, R. Ramiro Barcelos 910, Porto Alegre, RS 90570-001, Brazil

**Keywords:** Cardiac sarcoidosis, Clinically isolated cardiac sarcoidosis, Multimodality imaging, Cardiac magnetic resonance, ^18^F-FDG PET, Case report

## Abstract

**Background:**

Cardiac sarcoidosis remains a major diagnostic challenge when myocardial inflammation occurs in the absence of clinically active extracardiac disease. In this setting, non-invasive tissue characterization and metabolic imaging may provide critical incremental value over conventional structural assessment.

**Case summary:**

A 62-year-old woman with progressive exertional dyspnoea and long-standing ventricular ectopy underwent multimodality evaluation. Echocardiography showed mild biventricular systolic dysfunction, reduced global longitudinal strain, and focal basal-to-mid septal abnormalities. Coronary computed tomography (CT) angiography excluded obstructive coronary artery disease and showed inactive pulmonary granulomatous sequelae. Cardiac magnetic resonance demonstrated mild-to-moderate biventricular dysfunction and patchy non-ischaemic late gadolinium enhancement in the basal-to-mid septum with elevated native T1 values but no T2 evidence of oedema. ^18^F-fluorodeoxyglucose positron emission tomography/CT subsequently revealed focal and multifocal myocardial uptake on a suppressed background, confirming active myocardial inflammation without extracardiac hypermetabolic disease. Immunosuppressive therapy was initiated, and guideline-directed heart failure therapy was optimized.

**Discussion:**

This case illustrates how stepwise multimodality imaging can establish the diagnosis of active cardiac sarcoidosis, provide precise non-invasive tissue characterization, distinguish active inflammation from fibrosis, and directly guide immunosuppressive treatment when extracardiac disease is clinically silent.

Learning points

- Cardiac sarcoidosis should be suspected in patients with ventricular arrhythmias and unexplained septal abnormalities, despite absent extracardiac activity.
- In patients with non-ischaemic late gadolinium enhancement without T2 oedema on cardiac magnetic resonance, ^18^F-fluorodeoxyglucose positron emission tomography is essential to distinguish active inflammation from scar.
- Early diagnosis is essential, as prompt treatment may prevent progression and reduce the risk of heart failure or sudden cardiac death.

## Introduction

Cardiac sarcoidosis is an under-recognized inflammatory cardiomyopathy associated with ventricular arrhythmias, conduction abnormalities, and heart failure.^1,2^ Clinically isolated cardiac sarcoidosis (CICS) is a challenging phenotype, as myocardial involvement may occur without clinically active extracardiac disease.^1,2^ Because endomyocardial biopsy has limited sensitivity due to the patchy distribution of granulomatous infiltration, multimodality imaging has become central to non-invasive diagnosis, activity assessment, and therapeutic guidance.^1,3^ We report a case of active CICS with inactive pulmonary sequelae in which echocardiography, cardiac magnetic resonance (CMR), and ^18^F-fluorodeoxyglucose positron emission tomography (^18^F-FDG PET) were pivotal to diagnosis and management within a stepwise multimodality workflow, highlighting the value of an integrated imaging pathway for timely decision-making in routine care.

## Summary figure

Multimodality diagnostic workflow in clinically isolated cardiac sarcoidosis. Timeline of symptoms and investigations. Echocardiography demonstrates regional septal strain impairment. Cardiac magnetic resonance shows non-ischaemic late gadolinium enhancement in the basal-to-mid septum. ^18^F-fluorodeoxyglucose positron emission tomography confirms active myocardial inflammation in the absence of extracardiac uptake, leading to immunosuppressive treatment initiation and follow-up imaging.

**Figure:**
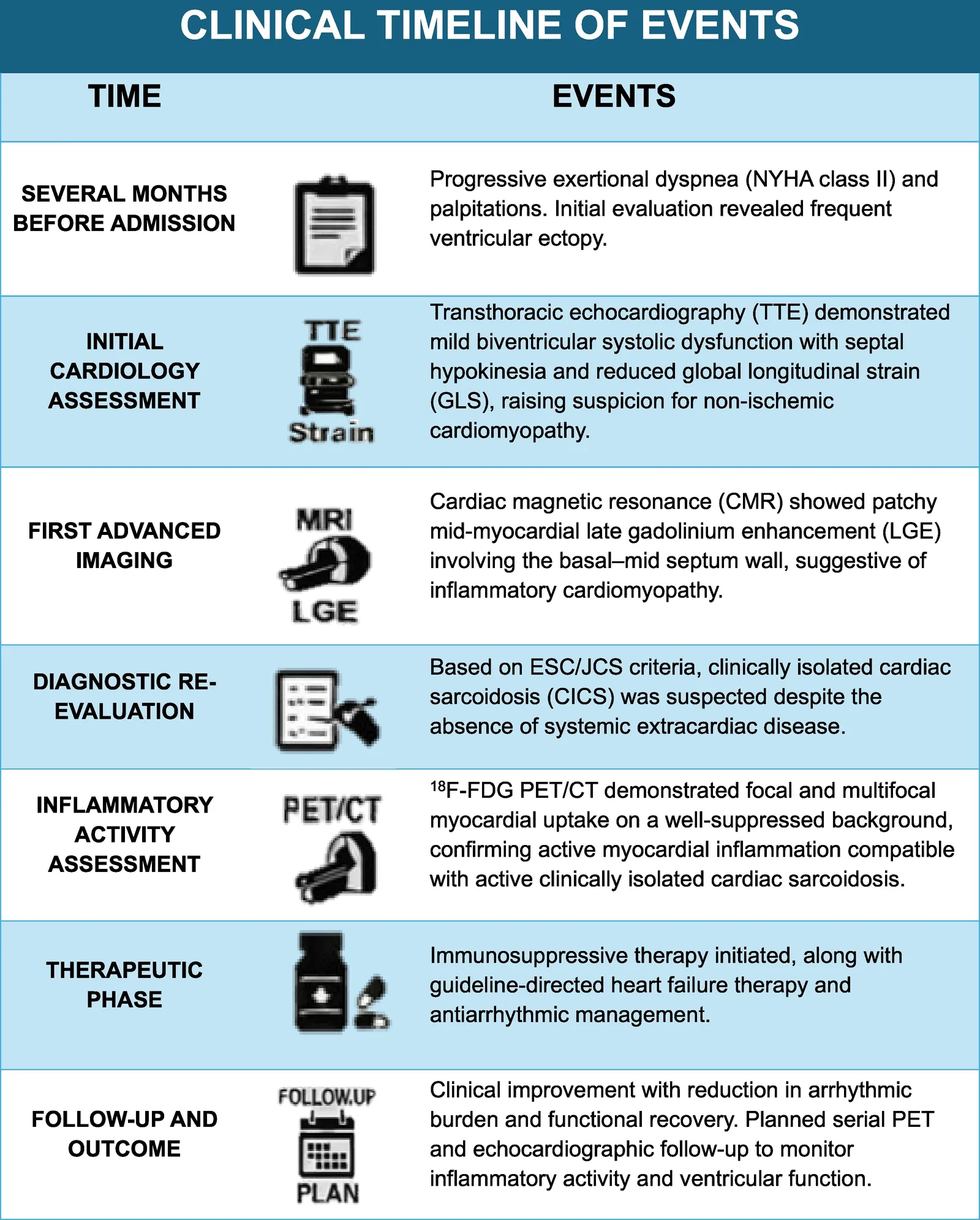


## Case presentation

A 62-year-old woman with a 3-year history of ventricular ectopy presented with progressive dyspnoea over the preceding 12 months (New York Heart Association [NYHA] class II). She had no prior systemic disease and denied syncope, presyncope, and sustained palpitations. On examination, blood pressure was 134/85 mm Hg and heart rate was 82 bpm, without signs of heart failure. High-sensitivity troponin, inflammatory markers, and N-terminal pro-B-type natriuretic peptide (NT-proBNP) were normal. Twenty-four-hour Holter monitoring showed sinus rhythm, with no atrial fibrillation, significant pauses (>2.5 s), or ventricular tachycardia; the combined ventricular and supraventricular ectopic burden was 5%, without symptom correlation (Supplementary material online, *Figure S1*).

**Figure 1:**
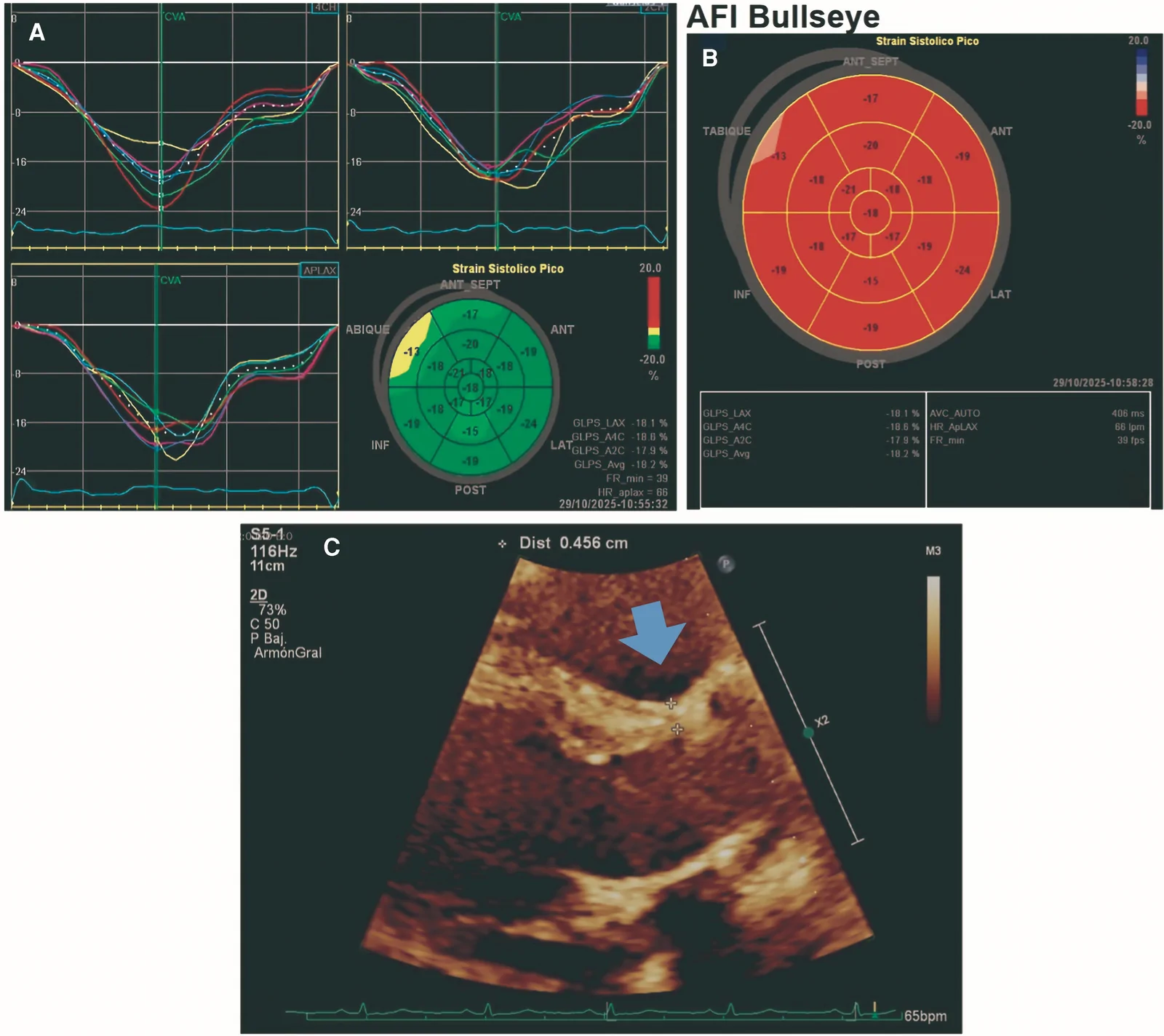
Speckle-tracking echocardiography. *(A)* Longitudinal strain curves showing a global longitudinal strain of −18%, consistent with subclinical to mild left ventricular dysfunction and a regional pattern suggestive of patchy myocardial involvement. *(B)* Polar map (‘bull’s-eye’) demonstrating regional reduction of longitudinal strain, predominantly involving the basal and mid-septal segments. *(C)* Basal thinning of the interventricular septum is also evident (arrow).

**Figure 2:**
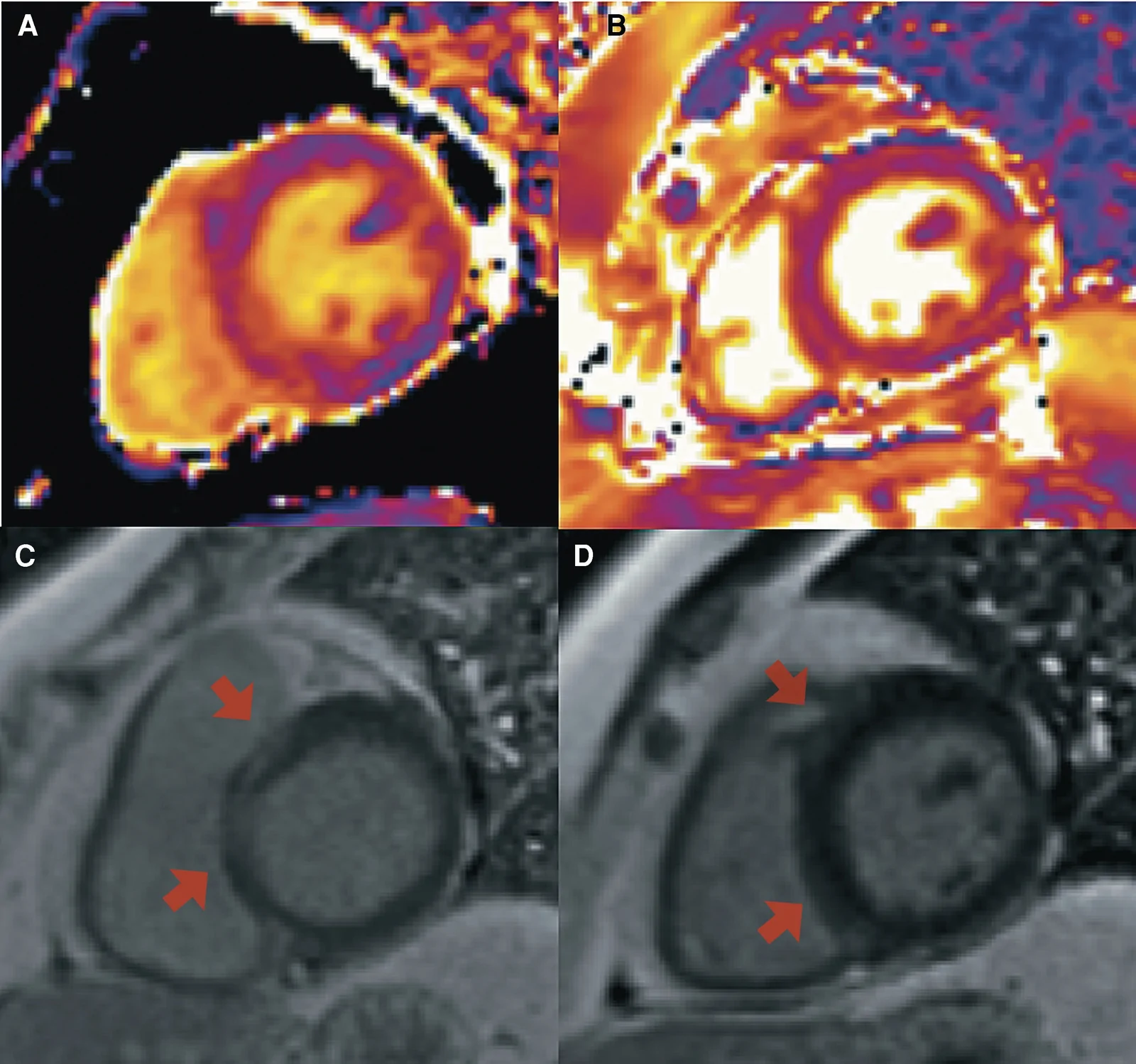
Cardiac magnetic resonance—non-ischemic late gadolinium enhancement. *(A)* T2-weighted turbo spin-echo sequences showing no evidence of myocardial edema. *(B)* Late gadolinium enhancement sequences demonstrating intramyocardial enhancement in the basal septal wall segment (arrows). *(C)* Quantitative myocardial T1 mapping showing evidence of interstitial fibrosis in the basal septal wall segment. *(D)* Quantitative myocardial T2 mapping showing no evidence of myocardial edema.

Transthoracic echocardiography showed normal left ventricular size with mild biventricular systolic dysfunction (left ventricular ejection fraction [LVEF] 52%), basal-to-mid septal hypokinesia, reduced global longitudinal strain (GLS) of −18%, abnormal apical and basal torsion, and basal-to-midventricular septal thinning (*Figure 1*; Supplementary material online, *Video S1*). Coronary computed tomography (CT) angiography excluded obstructive coronary artery disease (Supplementary material online, *Figures S3* and *S4*), and showed calcified pulmonary granulomas (Supplementary material online, *Figure S5*). CMR revealed mild-to-moderate biventricular dysfunction (LVEF 47%; right ventricular ejection fraction 43%) and patchy non-ischaemic late gadolinium enhancement (LGE) in the basal-to-mid septum, with elevated native T1 values consistent with interstitial fibrosis and no T2 evidence of oedema (*Figure 2*; Supplementary material online, *Video S2*).

**Figure 3:**
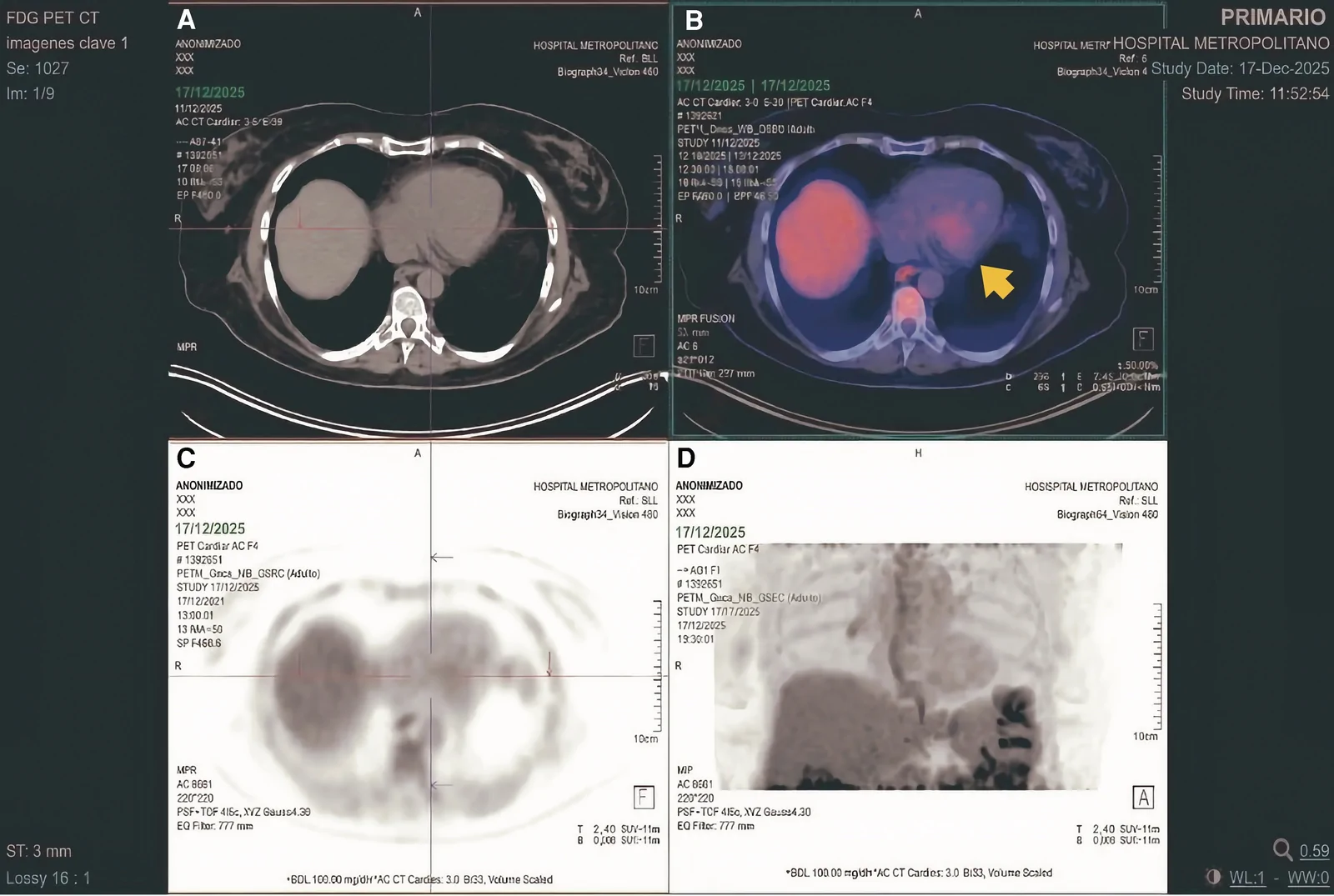
^18^F-fluorodeoxyglucose positron emission tomography demonstrating active myocardial inflammation. *(A)* Axial non-contrast computed tomography image showing preserved cardiac morphology without structural extracardiac abnormalities. *(B)* Fused positron emission tomography/computed tomography axial image demonstrating focal and multifocal myocardial ^18^F-fluorodeoxyglucose uptake predominantly involving the interventricular septum and left ventricular segments (arrow). *(C)* Axial positron emission tomography image confirming non-coronary patterned myocardial hypermetabolism on a well-suppressed background (arrows). *(D)* Whole-body maximum-intensity-projection image showing absence of extracardiac hypermetabolic lesions.

^18^F-FDG PET/CT, performed after appropriate metabolic preparation, demonstrated focal and multifocal myocardial uptake predominantly in the interventricular septum on a suppressed background, confirming active myocardial inflammation, with no extracardiac hypermetabolic lesions (*Figure 3*).

Given the absence of syncope, sustained ventricular arrhythmias, high-grade atrioventricular block, or severe left ventricular dysfunction, electrophysiological study, implantable loop recorder, or prophylactic implantable cardioverter-defibrillator implantation were not indicated according to current Heart Rhythm Society (HRS) expert consensus recommendations.^4,5^

At 2-month follow-up, the patient reported improvement in exertional dyspnoea, with NYHA functional class improving from II to I. Immunosuppressive therapy had been initiated with deflazacort 30 mg daily for 1 month, followed by discontinuation. Methotrexate 17.5 mg subcutaneously once weekly, together with folic acid 5 mg weekly, was introduced as steroid-sparing therapy and is planned to be continued for 3 months. Propranolol 10 mg three times daily was maintained for control of ventricular arrhythmias, while empagliflozin 10 mg daily was continued as part of guideline-directed heart failure therapy. Repeat Holter monitoring demonstrated a reduction in ventricular arrhythmia burden from 5% to 1%, with no high-grade atrioventricular block, heart failure events, steroid-related major adverse effects, or other unexpected events during follow-up (Supplementary material online, *Figure S2*).

This case reflects an intermediate-risk cardiac sarcoidosis phenotype, with CMR scar, active ^18^F-FDG uptake, and mild biventricular dysfunction, but without major high-risk arrhythmic or conduction features. Early clinical improvement is reassuring; however, close follow-up is warranted, and repeat ^18^F-FDG PET/CT is planned in 3 months to reassess inflammatory activity.^3,5,6^

## Discussion

Cardiac sarcoidosis should be suspected when otherwise unexplained regional ventricular dysfunction, septal thinning, and ventricular arrhythmias coexist, even in the absence of clinically active extracardiac sarcoidosis. Current diagnostic frameworks recognize that myocardial disease may dominate the clinical presentation and that endomyocardial biopsy has limited sensitivity because of the patchy distribution of granulomatous infiltration. In this context, multimodality imaging becomes central not only for detection, but also for biological phenotyping and therapeutic decision-making.^1-3,5^

In the present case, echocardiography provided the first clue by identifying a regional mechanical abnormality disproportionate to the global degree of ventricular dysfunction with basal-to-midventricular septal thinning.^5,7^ CMR then defined a highly suggestive structural phenotype and patchy non-ischaemic LGE involving the septum, a distribution repeatedly associated with cardiac sarcoidosis.^8,9^ However, CMR alone could not determine whether this represented inactive scar or ongoing immune-mediated injury. The incremental contribution of ^18^F-FDG PET was therefore decisive: Focal myocardial hypermetabolism on a suppressed background confirmed active inflammation and directly supported initiation of immunosuppressive therapy.^3,5,6^

The main differential diagnoses included inflammatory cardiomyopathy, cardiac sarcoidosis, infectious cardiomyopathy, amyloidosis, Fabry disease, hypertrophic cardiomyopathy, arrhythmogenic cardiomyopathy, and chronic ischemic scar. Infectious cardiomyopathy became less likely after laboratory and serological evaluation showed no evidence of active infection. Obstructive coronary artery disease was excluded by coronary CT angiography. The combination of basal-to-mid septal thinning, patchy non-ischaemic septal LGE, and abnormal regional strain strongly favoured a sarcoid pattern.^7–9^ Because CMR showed fibrosis without oedema, ^18^F-FDG PET/CT was performed to assess residual inflammatory activity and confirmed active cardiac sarcoidosis despite the absence of clinically active extracardiac disease.^3,5,6^

A major strength of this case is the concordant use of strain imaging, CMR, and ^18^F-FDG PET/CT to establish a phenotype-based diagnosis. Endomyocardial biopsy was not considered indispensable because unguided biopsy has an ≈20% sensitivity in cardiac sarcoidosis owing to the patchy myocardial distribution of disease and carries procedural risk, whereas PET directly identified active myocardial inflammation.^5^

## Conclusion

This case highlights that active cardiac sarcoidosis may present as a predominantly cardiac inflammatory phenotype despite clinically silent extracardiac disease. When echocardiography and CMR suggest a sarcoid pattern, ^18^F-FDG PET provides decisive incremental value by distinguishing active inflammation from fibrosis and by guiding immunosuppressive treatment.^2,3,5^

## Patient perspective

The patient expressed relief at finally receiving a clear diagnosis and appreciated the non-invasive diagnostic approach and defined treatment plan.

## Supplementary Material

ytag545_Supplementary_Data

## Data Availability

The data underlying this article are available within the article and its Supplementary material. No additional datasets were generated or analysed.

## Lead author biography

Leonardo Briceño, MD is a cardiologist and advanced cardiac imaging specialist based in Loja, Ecuador. He completed his cardiology residency at the University of Buenos Aires. He holds a Master’s degree in Advanced Cardiac Imaging from the Universidad San Antonio de Murcia (UCAM, Spain) and completed formal postgraduate university training in Echocardiography and Cardiac Doppler at Instituto Universitario CEMIC (Argentina). He holds a University Diploma in Nuclear Cardiology from the Pontificia Universidad Católica Argentina. He is currently a staff cardiologist at Hospital Santa Inés and SOLCA. His academic interests include multimodality cardiac imaging, cardio-oncology, and nuclear cardiology.

## Notes

**Consent:** The authors confirm that written consent for submission and publication of this case report including images and associated text has been obtained from the patient, in line with COPE guidance.
