# Supplementary material for: Active clinically isolated cardiac sarcoidosis with inactive pulmonary sequelae: incremental diagnostic value of multimodality imaging: a case report
Source: Eur Heart J Case Rep. 2026 Aug 17;10(9):ytag545. doi: 10.1093/ehjcr/ytag545 (PMC13542721; doi:10.1093/ehjcr/ytag545)
Supplement: Supplementary file 1 [file ytag545_supplementary_data.zip › Supplementary captions.docx]

**SUPPLEMENTARY IMAGES**

**Supplementary image 1: Baseline Holter Monitoring**

Twenty-four-hour Holter recording showing sinus rhythm as the predominant rhythm during the study. Mean heart rate was 88 bpm with preserved heart rate variability. Moderate ventricular ectopic burden was observed (5%, 6326 premature ventricular complexes) occurring as couplets, bigeminy, and trigeminy **(orange arrows)**. Low supraventricular ectopic burden was also present (0.1%, 116 premature atrial complexes). No pauses or sustained arrhythmias were detected.

Alt text: Holter monitor strip showing a rhythm with visible ventricular complexes marked with an orange arrow.

**Supplementary image 2: Follow-up Holter Monitoring After Antiarrhythmic Therapy**

Twenty-four-hour Holter monitoring performed after initiation of propranolol therapy demonstrated sustained sinus rhythm throughout the recording period. A total of 117,083 beats were analysed, with a mean heart rate of 81 bpm (maximum 135 bpm, minimum 53 bpm). Heart rate variability was preserved (standard deviation of normal-to-normal intervals [SDNN] 229ms; percentage of successive RR intervals that differ by more than 50 ms [pNN50] 5.28%). The ventricular ectopic burden was markedly reduced, with only 124 premature ventricular complexes (<1% of total beats) **(orange arrows)**. A very low supraventricular ectopic burden was also observed. No episodes of atrial fibrillation, significant pauses (>2.5 s), or sustained arrhythmias were detected.

Alt text: Holter monitor strip showing reduced ventricular complex burden after therapy, marked with an orange arrow.

**Supplementary image 3: Coronary Artery Calcium Score.**

Non-contrast cardiac CT demonstrating a coronary artery calcium score of 0 Agatston units, indicating a very low probability of significant coronary atherosclerosis. Incidental findings include focal centrilobular emphysema, scattered pulmonary cysts, and multiple calcified pulmonary granulomas.

Alt text: Non-contrast chest computed tomography scan showing an absence of coronary calcification alongside incidental pulmonary cysts and calcified granulomas.

**Supplementary image 4. Coronary CT Angiography**

Three-dimensional volume-rendered reconstruction demonstrating normal epicardial coronary arteries without evidence of coronary atherosclerosis (Coronary Artery Disease Reporting and Data System [CAD-RADS] 0). The ascending aorta and pulmonary artery are within normal limits, and left ventricular dimensions, wall thickness, and systolic function are preserved, with an estimated left ventricular ejection fraction of approximately 60%.

Alt text: Three-dimensional volume-rendered reconstruction of a coronary CT angiography showing normal and patent coronary arteries.

**Supplementary image 5: High-Resolution Chest Computed Tomography (HRCT)**

Axial high-resolution CT image of the chest demonstrating multiple small bilateral pulmonary cysts measuring approximately 4–10 mm, predominantly involving the upper lobes, findings consistent with mild cystic lung disease. In addition, multiple bilateral calcified pulmonary granulomas are observed **(green arrows)**, compatible with sequelae of prior granulomatous disease. No evidence of pulmonary consolidation, ground-glass opacities, masses, or pleural effusion is identified on this study.

Alt text: Axial high-resolution chest CT scan showing multiple small bilateral pulmonary cysts and calcified granulomas in the upper lobes.
